# Translation and cultural adaptation of the revised foot function index for the Portuguese language: FFI-R Brazil

**DOI:** 10.1590/1516-3180.2017.0183030817

**Published:** 2017-12-07

**Authors:** Liu Chiao Yi, Ana Carolina Camacho Cabral, Danilo Harudy Kamonseki, Elly Budiman-Mak, Milena Carlos Vidotto

**Affiliations:** I PhD. Professor, Department of Human Movement Sciences, Universidade Federal de São Paulo (UNIFESP), Campus Baixada Santista, Santos (SP), Brazil.; II Undergraduate Student, Physiotherapy Course, Universidade Federal de São Paulo (UNIFESP), Campus Baixada Santista, Santos (SP), Brazil.; III Postgraduate Student, Universidade Federal de São Paulo (UNIFESP), Campus Baixada Santista, Santos (SP), Brazil.; IV PhD. Professor, Medicine Department, Loyola University of Chicago, Chicago, United States.

**Keywords:** Foot, Translations, Surveys and questionnaires, Outcome assessment (health care)

## Abstract

**BACKGROUND:**

The revised foot function index (FFI-R) is used to evaluate the functionality of patients with conditions that affect the feet. The objective here was to produce the Brazilian Portuguese version of this index.

**DESIGN AND SETTING:**

Translation and validation study conducted at the Federal University of São Paulo, Brazil.

**METHODS:**

The translation and cultural adaptation process involved translation by two independent translators, analysis by an expert committee, back translation into the original language, analysis by the expert committee again and a pretest. The Portuguese-language version was administered to 35 individuals with plantar fasciitis and metatarsalgia to determine their level of understanding of the assessment tool.

**RESULTS:**

Changes were made to the terms and expressions of some original items to achieve cultural equivalence. Terms not understood by more than 10% of the sample were altered based on the suggestions of the patients themselves.

**CONCLUSION:**

The translation and cultural adaptation of the FFI-R for the Portuguese language were completed and the Brazilian version was obtained.

## INTRODUCTION

Musculoskeletal injuries in the ankle and foot cause functional limitations that have a negative impact on quality of life.^1^ Classification of the degree of dysfunction is fundamental for characterization of patients’ status and enables quantification of the effect of treatment.^2^ The main assessment tools used to evaluate the functionality of the feet, such as the foot function index (FFI), foot and ankle outcome score (FAOS), foot health status questionnaire (FHSQ) and Manchester foot pain and disability index (MFPDI), were developed in the English language.^3^^,^^4^ For these assessment tools to be used in different countries with different languages, it is necessary to perform translation and cultural adaption and to test the psychometric properties of the adapted tools.^5^

The FFI is considered to be one of the main assessment tools for evaluation of the functionality of the ankle and foot, because all its psychometric properties have been validated.^1^^,^^6^^,^^7^ Subsequently, adjustments and new domains were added to broaden its scope, thereby creating the revised foot function index (FFI-R).^8^ In this version, the visual analogue scale (VAS) was replaced with a Likert scale. The domains and items of the original questionnaire were maintained and others regarding psychosocial characteristics were added. The FFI-R has five domains containing 68 items, with questions relating to pain (11 items), stiffness (8 items), problems (20 items), activity limitation (10 items) and social issues (19 items).^8^

Because of the importance of standardization when using evaluation measurements, questionnaires developed in foreign languages need to be translated and their psychometric properties evaluated, to create equivalence between studies. This process makes it possible for physicians and other professionals working in a given field to obtain a reliable tool for patient evaluations. Thus, the FFI-R can become available for assessing patients with foot and ankle musculoskeletal disorders.

The FFI has been translated and validated for use in several countries, such as Germany, Spain, France, China and Brazil.^9^^,^^10^^,^^11^^,^^12^^,^^13^^,^^14^ However, the revised version has not yet been translated and culturally adapted to any foreign language based on its original version.

## OBJECTIVE

The aim of the present study was to translate and culturally adapt the revised foot function index to the Brazilian Portuguese language.

## METHODS

**Figure 1.**
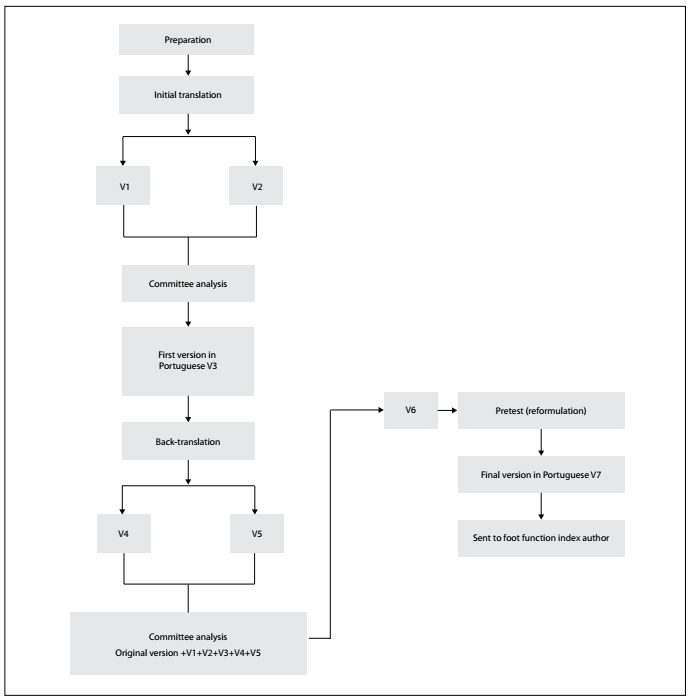
Flowchart of the study.

Thirty-five patients participated in this study: the first phase involved 20 volunteers and the second phase involved 15 other volunteers with plantar fasciitis and metatarsalgia. The participants were recruited through announcements in the printed and digital media and through verbal invitation. Their mean age was 25.2 years (range: 18 to 57 years) and females accounted for 57% of the sample. With regard to schooling, 12% had completed higher education and 80% were still studying. This investigation received approval from the human research ethics committee of the institution in which it was conducted (ethics committee no. 327.129) and all the participants signed a free and informed consent statement. The authorization for the use of the FFI-R was obtained from the original authors through electronic mail (Figure 1).

The translation and cultural adaption of the FFI-R followed the method described by Beaton et al.^15^ and the Guidelines for Reporting Reliability and Agreement Studies (GRRAS) were used:^16^

1. Translation;
2. Analysis by an expert committee;
3. Backtranslation into the original language;
4. Analysis by the expert committee again; and
5. Pretest.

The FFI-R was translated into Portuguese by two Brazilian professional translators who were fluent in English. The translators were informed regarding the objective of the study and the two versions of the translation thus produced (V1 and V2) were developed independently.

The two translations and the original questionnaire were compared and discussed by the members of the expert committee, in order to reach a consensual version in Portuguese that maintained the fundamental characteristics of the original questionnaire, thus forming V3. In the backtranslation phase, V3 was translated back into English by two translators whose native language was English and who had no access to the original questionnaire. These versions (V4 and V5) were shown to the expert committee. The committee discussed the differences between all the versions created and the original questionnaire. Inadequate or ambiguous items were altered, changes were suggested and equivalences were determined, regarding the meanings of words, idiomatic equivalence (interpretation of colloquialisms), cultural equivalence (to ensure that the practices mentioned in the questionnaire were common to the new culture to which it would be administered) and conceptual equivalence (to determine the cultural importance of the situations presented in the questionnaire).

Sentences were rewritten as necessary until a consensual version of the index in Portuguese had been obtained. This version was then used in the pretest, which was divided into two parts: V6-1 and V6-2. V6-1 was administered to 20 patients to determine the understanding of the questions. The researcher read aloud the content of the questionnaire to each participant, who then made suggestions if any items required a change (Table 1).

**Table 1.**
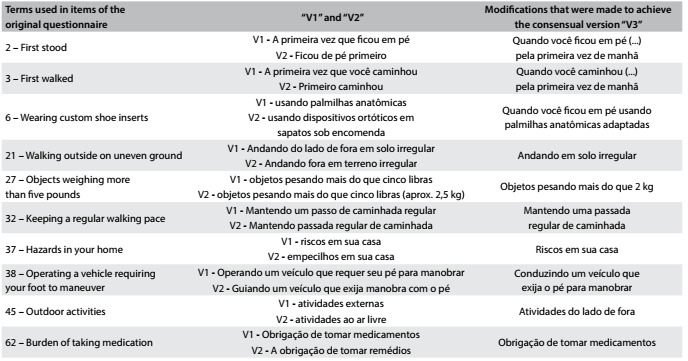
Translation phase. Changes in “V1” and “V2” to obtain “V3”

V1 = translator 1 version; V2 = translator 2 version; V3 = consensual version in Portuguese after translation phase.

Items that did not achieve a level of understanding that exceeded 90% of the volunteers were rewritten, which thus created V6-2. This new version was administered to another 15 patients, who underwent the same procedures as were used for V6-1, until all items in the questionnaire were understood by more than 90% of the patients, which led to the final V7 version. This version was sent to the author of the original FFI-R, who did not suggest any changes.

## RESULTS

In the translation phase, the two versions of the translated questionnaire (V1 and V2) were compared and were used to create the first consensual version (V3) (Table 1).

In the backtranslation phase, V3 and the backtranslated versions (V4 and V5) were analyzed and compared with the original questionnaire in English in order to develop V6. This stage involved grammatical, semantic and idiomatic changes for cultural adaptation of the questionnaire while maintaining the objective of each item (Table 2).

**Table 2.**
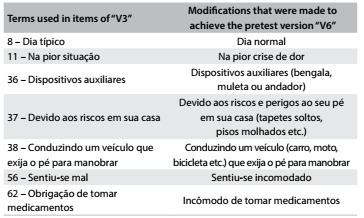
Back translation phase. Changes in “V4” e “V5” to obtain “V6

V3 = consensual Portuguese version of V1 and V2; V6 = final Portuguese version, after analyzing the original version, V3, V4 and V5.

In the pretest phase, items that were not understood were altered based on suggestions provided by the patients, thus leading to the final version of the questionnaire in Portuguese (Table 3).

**Table 3.**
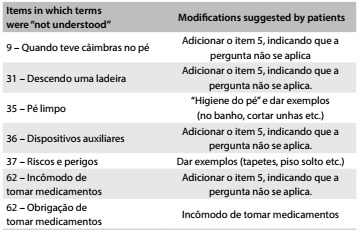
Modifications to the pretest phase that were made

## DISCUSSION

The translation and cultural adaptation process on the revised foot function index, for use in Portuguese was performed and the Portuguese language version for use in Brazil was achieved. The cultural adaption process for the FFI-R^8^ followed the method proposed by Beaton et al.^15^ Several other questionnaires that have been translated and validated for the Portuguese language have followed this model, such as the FFI,^9^ WOMAC (Western Ontario and McMaster Universities)^17^ and FAOS.^18^ The questionnaire was administered to a greater number of young, physically active women, which was similar to the method that had been used for the original questionnaire.^8^ In the initial phase of translation into the Portuguese language, the term “five pounds” was replaced with 2 kg by the expert committee, since this is the measurement unit for mass that is used in Brazil, thereby allowing patients to correlate the measurement unit with the mass of common objects used in everyday life.

In the back translation, question 35 was discussed during the analysis by the committee because it had been translated in a literal fashion. The expression “keeping your foot clean” in English is quite precise and specific, but when translated into Portuguese, this resulted in “*mantendo o pé limpo*”, which caused a lack of understanding. Nevertheless, the committee suggested that this question should be kept in the same format for the pretest phase, to test its clarity in practice. In the first phase of the pretest, approximately 50% of the interviewees had doubts about the meaning and the expression *“mantendo a higiene do pé”* [maintaining the hygiene of the foot] was suggested. After this change, there were no longer any doubts in the second phase of the pretest.

In the backtranslated version of item 62, the committee thought that the original word “burden” did not have the same meaning as the backtranslated word (obligation, from *“obrigação”*). Therefore, the word in the Portuguese version was replaced with “*incômodo*” [inconvenience], to maintain the same idea as in the original word.

With regard to the term *“rigidez”* [stiffness], the interviewees defined it as passive resistance of muscles, tendons, ligaments and fascia, since rigidity is a mechanical property relating to resistance of these tissues to deformation in the absence of muscle contraction.^19^

In the original questionnaire, the Likert scale has a fifth option (“does not apply”) for some items. In the second phase of the pretest, this option 5 was added to more items, as shown in Table 3, since these items did not apply to the majority of the individuals interviewed. In the sample, 80% of the participants were students at a public university and 12% had completed their university education. Thus, there was no considerable difference with regard to the level of understanding of the questionnaire among the interviewees.

Original questionnaires in English that have been validated for use in Brazil are generally submitted to a pretest process to obtain the final version in Portuguese, as well as to evaluate the psychometric properties, such as reliability and validity, which are applied in interview form. This type of application has been used in Brazil because of the profile of the populations evaluated during the process, most of whom are recruited from public clinics and hospital services. Although the use of two pretest phases is not commonly found in the literature, important questionnaires that have frequently been cited, such as the SF-36,^20^ FHSQ^4^ and WORC,^19^ have also used this model. Pretesting is an important phase in the cultural adaptation process, since it demonstrates patients’ interpretation of the items in a questionnaire. Thus, two pretest phases were used for the FFI-R to ensure that the final version would be understood by more than 90% of the patients^21^^,^^22^ and that the questionnaire would be culturally adapted to the Brazilian population. The psychometric properties of the FFI-R are currently in the test phase to validate the questionnaire for use in Brazil.

## CONCLUSION

The translation and cultural adaptation of the FFI-R for the Portuguese language were completed and the Brazilian version was obtained.
